# Commencement R. M. C.

**Published:** 1868-02-01

**Authors:** 


### *EDITORIAL.*

---

BACK NOS. WANTED.—*Owing to the large increase in our subscription list, although it was supposed a sufficient number of copies had been provided, we are entirely out of copies of the* JOURNAL *for the months of* JANUARY, JUNE, SEPTEMBER, OCTOBER, *and* NOVEMBER, 1867, *Vol.* XXIV. *We will pay twenty-five cents each for copies of either of these, in cash or by credit on the present volume, as desired. Will our friends who do not file their numbers oblige us by responding?*

### *Commencement R. M. C.*

The exercises of the Twenty-Fifth Annual Commencement of Rush Medical College will take place on Wednesday the 6th inst., at the lower lecture-room of the College, at half-past 7 o'clock, p. m.

The Valedictory Address will be pronounced by Prof. R. L. Rea. Diplomas will be awarded to the largest graduating class in the history of the Institution. The profession are cordially invited to be present.

